# The impact of single versus multiple recasts on L2 learners' implicit and explicit knowledge

**DOI:** 10.1016/j.heliyon.2019.e01748

**Published:** 2019-05-24

**Authors:** Mohammad Hassanzadeh, Fahimeh Marefat, Arezoo Ramezani

**Affiliations:** a Vali-e-Asr University of Rafsanjan, Iran; b Allameh Tabataba'i University, Iran

**Keywords:** Linguistics, Education, Psychology

## Abstract

Recasts have been the object of extensive theoretical and empirical investigation in second language acquisition research since the mid-1990s. Despite being acknowledged to have a facilitative effect on second language (L2) learning, the extent of their acquisitional contribution is a matter of controversy. This study examined the effectiveness of single and multiple recasts (SR & MR) on the acquisition of a planned target structure, as represented on the learners' implicit and explicit knowledge. The participants were three intact groups of English as a foreign language (EFL) learners at a language institute in Iran. The two experimental conditions received respective recasting on errors of English unreal conditionals. All groups – including a third control condition – were then tested via three discrete tasks aimed at measuring their implicit and explicit knowledge: an *elicited oral imitation task*, a *timed grammaticality judgment task*, and an *untimed grammaticality judgment task*. The results revealed that both groups exhibited improvements on both measures along immediate and delayed occasions. However, their performance over the two implicit knowledge measures yielded dissimilar outcomes. Pedagogically speaking, it must be realized that recasting would not necessarily lead to acquisition unless L2 teachers become more conscious of where and how to orchestrate them.

## 1 Introduction

Corrective feedback (CF) and the extent of its effectiveness in improving L2 learners' grammatical accuracy has been one of the principal concerns of second language acquisition (SLA) research over the recent years. Even though most L2 teachers and educators, both in theory and practice, corroborate the importance of feedback provision, disagreements abound as to what type of CF would fare better for different learners and different linguistic features (Ammar and Spada, 2006; Li, 2010; Yang and Lyster, 2010). As a seminal type of CF, recasts consist of the teacher's changing of all or part of the learner's utterance that includes at least one error within the situation of a task-based activity in the classroom environment (Sheen, 2006). Recasts can take many different forms and perform copious functions, rendering their definition rather far from straightforward. Doughty and Varela (1998) observed that recasts put emphasis on the error and a reformulation of that error. Similarly, Long (1996) explained that a recast rephrases an erroneous learner utterance 'while still referring to its central meaning' (p. 434). Given the vast array of views on recast, as Ellis and Sheen (2006) argued, it may well be that we are not targeting a uniform concept, but one which is open to interpretation, ranging along an implicit-explicit continuum. Recasts offer both negative feedback and positive input to the learners' problem in production (Leeman, 2003), thereby catering to acquisition largely for being implicit and unobtrusive (Long, 1996). In contrast, explicit forms of correction involve treating language as an object and interrupting the flow of communication which might get in the way of form-function mapping. Last, recasts are learner-centered in that their usage is associated with the meaning that the learners are trying to communicate (Mackey, 2007). Despite its observed merits, recasting remains a controversial issue among many SLA researchers. Several empirical studies demonstrated that it is facilitative of several linguistic forms in L2 development: Tense consistency in English (Han, 2002), noun-adjective gender agreement in Spanish (Leeman, 2003), acquisition of English noun plurals (Zhuo, 2010), and pronunciation teaching (Saito, 2015), among others. Nonetheless, there are studies that suggest that recasts, despite the strong theoretical claim regarding their acquisitional potential, are not necessarily better than other types of CF such as prompts or explicit corrective strategies (Lyster, 2004; Ammar and Spada, 2006; Yang and Lyster, 2010). Given the indirect and multifunctional nature of recasts, it is not obvious to what extent they can smooth the way for acquisition. Learners have been observed to have difficulty responding to them. The problem is compounded when learners do not notice the recast, on the grounds that naturally, interlocutors also repeat the learners' correct utterances; hence learners can never be sure whether the teacher has corrected their errors or has encouraged them for what they have correctly uttered (Lyster, 1998b). Batstone (2002) argued that learners sometimes find themselves in a dilemma between two simultaneous contexts of learning versus communication. In a communicative context where language is treated as a message, learners might not become aware of the recast's corrective force; whereas in a learning context where language is deemed as an object, they stand a better chance of noticing it. Although a number of studies (e.g., Mackey et al., 2000; Nabei and Swain, 2002) attempted to establish what learners attend to during recasts, there has been a dearth of research comparing the impact of various recast types. Furthermore, despite the fact that recasts have been a popular issue of investigation over the past two decades, relatively few studies to date have measured their effectiveness on implicit and explicit knowledge distinctively. Some evidence suggests that recasts may be more beneficial for implicit knowledge (Li, 2010). However, their effectiveness for explicit knowledge is less obvious (Ellis and Sheen, 2006).

## 2 Background

In this section, we will lead off by providing a more detailed description of the existing classifications surrounding recasts and the relevant lines of research in this area. Later, we will provide a background to what explicit and implicit types of knowledge are and the illuminating role that further empirical research into the impact of CF can play on these cognitive constructs.

### 2.1 Types of recast

Recasts can be variable in their features, especially with respect to their length, prosodic adjustment (such as stress or rising intonation) and the number of errors in question (Mackey, 2012). Hence, some types of recast may be more salient than others. Philp (2003) found that the length of recast and the number of changes therein were significant predictors of the learners' accurate recall of the correct form. Factors affecting or mediating the relationship between recasting and learning could be enumerated as the type of recast, the linguistic form, the context, language proficiency, learners’ developmental readiness, and other individual learner differences such as language anxiety (Sheen, 2008), gender (Ross-Feldman, 2007), working memory capacity (Goo, 2012; Révész, 2012), and language aptitude (Li, 2013; Sheen, 2008). On balance, recasts are deemed as implicit feedback because they are not introduced by phrases such as *you mean* …, *use this word* …, or *you should say* …. Contrary to many forms of CF, recasts are less direct and more subtle. Though widely recognized as implicit, they sometimes make the corrective force quite conspicuous; hence, it is easy to find examples of them that resemble explicit correction. The research literature on recasts is marked by a number of classifications, as illustrated below:

As can be seen in Table 1, there are good reasons why recasts have been so earnestly embraced by SLA researchers. First, they have been found to occur more frequently than any other kind of CF in both classroom and naturalistic settings (Lyster and Ranta, 1997; Panova and Lyster, 2002). Sheen (2004), in a study that compared the frequency of recasts in three contexts including immersion, communicative English as a second language (ESL), and English as a foreign language (EFL), discovered that on average, 60% of all feedback moves involved recasts. Second, studying recasts serves as a means of investigating issues of general theoretical pre-eminence in SLA, including positive evidence (Nicholas et al., 2001), and negative evidence (Leeman, 2003; Long, 2015). Moreover, they encourage interaction (Ellis and Sheen, 2006), and promote the saliency of target forms (Leeman, 2003; Loewen and Philp, 2006).

**Table 1:** Recast typology.

Source	Recast type	Definition
Doughty and Varela (1998); Nassaji (2009)	(a) Explicit	consists of repetition of a wrong utterance to draw the learner's attention followed by a recast to provide the contrastive L2 form.
	(b) Implicit	only involves a reformulation of a learner's non-target-like utterance into a target-like one.
Farrar (1992)	(a) Corrective	corrects a target error.
	(b) Non-corrective	does not correct but models a target.
Lyster (1998b)	(a) Declarative	the teacher reformulates all or part of the utterance with falling intonation and no additional meaning.
	(b) Interrogative	is the same as declarative recast except for incorporating a rise of intonation.
	(c) Confirmation of the original utterance	additional information is provided by incorporating the correct reformulation of all or part of the learner's utterance into a longer statement.
	(d) Additional information	there is extra information by incorporating the correct reformulation of all or part of a learner's utterance into a question.
Sheen (2006)	(a) Full	there is a reformulation of the whole erroneous utterance. only part of the erroneous utterance plus the error is repeated.
	(b) Partial	
Nelson et al. (1984)	(a) Simple	This will depend on whether the changes to the learner's wrong utterance are minimal (simple) or substantial (complex), and on the nature of the change; that is, whether it will include a substitution of the erroneous form, an addition, a deletion, or a reordering of the trigger utterance.
	(b) Complex	
Ellis and Sheen (2006); Kamiya (2015); Nassaji (2016)	(a) Extensive	is provided on a wide range of linguistic forms,
	(b) Intensive	is provided on a single preselected target structure

Another classification which has been opted for the current study is referred to as *single* and *multiple.* A single recast is embedded in a single teacher turn; typically operationalized as short, one-turn response moves following a learner's erroneous utterance (Loewen, 2009).

**Table:** 

**Example 1** (Ellis et al., 2001, p. 299)
L: I was in pub.
T: in the pub? *Single recast*
L: yeah and I was drinking beer with my friend.
T: which pub did you go to?

In Example 1, the teacher attempted to correct the student's error by a rise of intonation and using an interrogative sentence. In multiple recasts (example 2), however, the interlocutor repeats the recasts (Ellis and Sheen, 2006) as in below:

**Table:** 

**Example 2**
L: Kal told me your height is rather shorter.
T: Rather short. Rather short. *Multiple recasts*

By juxtaposing the above taxonomy, we would all perhaps agree with Ellis and Sheen (2006) that a recast is not a single genus. Given its ambiguous multidimensional function, learners may not always readily decipher the teacher's intent, i.e. correction.

### 2.2 Implicit and explicit knowledge

Implicit and explicit knowledge are two distinct types of knowledge which vary in terms of level of awareness. While in the former the knowledge is deployed without consciousness, in the latter some degree of awareness is required (DeKeyser, 2003; Rebuschat, 2013; Williams, 2009). Ellis (2005) and Ellis et al. (2009) developed multiple tests to measure implicit and explicit knowledge discretely. Measures such as the oral narrative task, timed grammaticality judgment task, and elicited oral imitation test performed under time pressure have been designed to gauge implicit knowledge. Alternatively, the untimed grammaticality judgment task and metalinguistic knowledge test are assumed to measure explicit knowledge without exerting any time pressure. In fact, time pressure is a critical factor in distinguishing between implicit and explicit knowledge. Nevertheless, it cannot necessarily restrict access to explicit knowledge as higher proficiency L2 learners may still access explicit knowledge in tasks that involve time limitation (Suzuki and DeKeyser, 2015) (see Ellis et al., 2009, for a comprehensive discussion of explicit and implicit systems of learning, knowledge, instruction and testing).

As acknowledged by Ellis and Loewen (2007), and Loewen and Philp (2006), although recasting has been investigated through much research, more substantive empirical studies employing measures of both implicit and explicit knowledge are needed to explore the ins and outs of this prevalent feedback strategy. Moreover, manipulating these two types of knowledge will more suitably demonstrate the impact of single and multiple recasts. Simply put, one might ask whether single and multiple recasts work better for implicit knowledge, explicit knowledge, or both. In order to contribute to L2 professional classroom practice, the current study attempted to examine the benefits of 'single' as well as 'multiple' recasting on the implicit and explicit knowledge of L2 learners' grammatical accuracy. Whereas in the former case (i.e., SR), language is used as a tool for achieving communication (learners may not be aware of the corrective force of recast), in the latter condition (i.e., MR), language is perceived as an object; that is, corrected forms are more conspicuous to the learners.

## 3 Method

To achieve our objectives, a quasi-experimental design consisting of three groups of EFL learners was adopted. Before delineating the methodology, let us present the research questions formulated for this study:

RQ1. To what extent is the acquisition of unreal conditionals affected by the provision of single and multiple recasts, as measured by tests of implicit knowledge?

RQ2., To what extent is the acquisition of unreal conditionals affected by the provision of single and multiple recasts, as measured by a test of explicit knowledge?

### 3.1 Participants

The sample consisted of three adult lower intermediate groups learning English in a reputable language institute known as Jahad Daneshgahi in Kerman, Iran. Data were culled from these Farsi-speaking male and female learners – aged between 17 and 40 (MR group, *N* = 28; SR group, *N* = 27; and control group, *N* = 22) – during a two-month period. The learners attended their regular classes three days a week, 90 minutes per session. One reason for choosing lower intermediate students was that these learners were more likely to notice recasts than their lower proficiency (i.e. elementary and beginner) peers (Philp, 2003). Moreover, the learners had not mastered the target structure (unreal conditionals) by the time of the experiment. The overall English proficiency level of the participants was measured by the Oxford Placement Test (Allan, 1992). Their curricular textbook was American English File, (volume 2, 2^nd^ edition, 2013). As for ethical considerations, the nature of the intervention was highly compatible with the educational objectives of the institute in question. Therefore, the researchers settled for the learners' informed verbal consent to take part in the experiment. Moreover, regarding ethical approval, the institutions (Vali-e-Asr University and Allameh Tabataba'i University) that the authors were affiliated with had waived the requirement to have such license.

### 3.2 Design and procedure

The study adopted a pretest – immediate posttest – delayed posttest design, with a control group. It is important to note that the treatments were conducted in six independent classes (three for SR and three for MR, respectively) in order to ease the way for the recasting procedure in the classroom environment. Each group participated in three tasks. An elicited oral imitation task (EOIT) coupled with a timed grammaticality judgment task (TGJT) were used to measure their implicit knowledge while an untimed grammaticality judgment task (UGJT) was used to measure their explicit knowledge (Ellis et al., 2009). Upon administering the pre-test, the treatments were provided, with the immediate posttest given shortly after the intervention, followed by the delayed posttest one week later. All participants in each group completed the TGJT and UGJT collectively and as for the EOIT, they individually completed the task. Before each task, the researcher (one of the authors of this study) explained the procedure to the participants. Then, some examples followed by certain practice items were given. As for the rating procedure, correct responses were coded as 1, and the incorrect responses as 0. The reliability index (Cronbach's alpha) for each immediate posttest task was calculated separately (TGJT = .70; EOIT = .68; and UGJT = .71).

### 3.3 Target structure

The target structure in the current study, was the English unreal conditional (hypothetical conditional). The motive behind choosing this target structure was its universally problematic quality to learners (Ellis, 2009). In addition, in keeping with minimizing the pre-existing effects of implicit or explicit knowledge, it was possible to ensure that the structure had not been taught to lower intermediate learners based on their institutional syllabus. The target structure implemented for the present research was the past tense form rather than past perfect, as it was assumed to be reasonably difficult for participants to learn (e.g., *If I were you, I would buy a car;* rather than: *If I had been you, I would have bought a car*). Three different example sentences of this structure were used in each of the tasks during the treatments in order to assure content validity: (a) were + would (e.g., *If I were a bird, I would fly to the sky*), (b) could + would (e.g., *If I could become a teacher, I would be happy*), and (c) verb + would (e.g., *If I found a million dollars, I would buy a big house*).

### 3.4 Treatment

During all treatment sessions, the researcher was present in the classes as an observer to ascertain whether the instructional procedure handled by the classroom teacher was going as intended. The teachers were briefed on what they had to do in advance. All the classes were conducted in a semi-circle seating style, which afforded the teacher to conveniently provide recasts for each participant when necessary. The treatment consisted of a sentence manipulation activity that elicited the unreal conditional orally from each participant. The sentences were adopted from an earlier study by Kamiya (2015), with trivial changes in content but not the structure. Out of eight sentences which were initially modeled by the teacher, participants were to produce one sentence each, using the unreal conditional in turn. When required, the teacher provided recasts. Any errors other than those of the target structure were ignored. The participants in the SR group received only single recasts and those in the MR group received multiple recasts for their errors of the unreal conditional. The participants in the control group received no recasts at all. That is to say, they uttered the eight unreal conditional sentences but did not receive any CF when errors occurred. Below are some examples of exchanges between the teacher (T) and students (S) during the treatment (erroneous utterances appear in boldface):

**Table:** 

**Example 3** (SR)
T: If I *were* a bird, I *would fly* to Australia because I would like to see the kangaroos. What would you do if you were a bird? (The teacher addressing one of the students).
S: If I *were* a bird, I **will** fly to China because I like Great Wall of China.
T: well, if I *were* a bird, I *would fly* to China because I like the Great Wall of China. (Rising intonation)

**Table:** 

**Example 4** (MR)
T: If I *couldvisit* the past, I *would go* back to the day when I was born.
S: If I **visit** the past …
T: If I *could visit* the past, *could visit* the past (Rising intonation, repeating twice and breaking down the communicative context into a learning context)
S: If I *couldvisit* the past, I **go** to …
T: I *would go, would go* …
S: If I *couldvisit* the past, I *would go* to my parents' wedding party.

**Table:** 

**Example 5** (No recast)
T: If I *were* a fish, I *would swim* around Hawaii because I would like to see the coral reef. What about you?
S: If I *were* a fish, I **will go** to the Persian Gulf to see the dolphins.

After nearly 30 minutes of interaction between the teacher and students, the researcher had the participants do the immediate posttest tasks. The order of posttest tasks was the same as the pretest. A week following the treatment and immediate post-testing, the subjects performed the tasks in the same order for the delayed posttest.

### 3.5 Instruments

As noted above, the instrumentation included three routine tests which are conventionally used by many SLA researchers interested in gauging learners' implicit and explicit knowledge.

#### 3.5.1 Timed grammaticality judgment task

The TGJT consisted of 24 items, including 12 unreal conditional sentences (six grammatical and six ungrammatical). The remaining 12 items were distractors (six grammatical and six ungrammatical). The order of the sentences was randomized across the three testing times. All the students sat in a teacher-fronted class facing the board. Then, the PowerPoint slides were set on automatic display. Upon viewing each sentence on the screen, the participants were to quickly judge whether it was grammatical by putting a tick or a cross on an answer-sheet that the researcher had provided earlier. The task was done under time pressure which is a crucial aspect of measuring implicit knowledge (Ellis, 2009). The time duration for each sentence amounted to 10 seconds which was determined through an earlier pilot study.

#### 3.5.2 Untimed grammaticality judgment task

The UGJT was a pen and paper test; a booklet consisting of single sentences printed on each page. The participants were to judge whether the sentences were grammatical or not. The participants were instructed not to go back to correct the previously completed items and that they were allowed to take as much time as they wanted. As in TGJT, the order of the sentences in UGJT was scrambled across the three tasks. The sentences in the UGJT were identical to those used at the TGJT, except for two distractor items.

#### 3.5.3 Elicited oral imitation task

The EOIT consisted of 24 opinion statements in total. Twelve of the items consisted of unreal conditional sentences, of those, six were grammatical and the other six ungrammatical. The remaining statements were distractors. To counterbalance the items, three versions of the tasks were randomly distributed. In a classroom, students were provided with headphones to be able to hear the statements more clearly. After they announced their readiness to do the task, the researcher played the audio tracks. After hearing each sentence, the participants had to indicate whether they agreed (yes), disagreed (no), or were unsure about the content of the statement. Then they had to repeat each sentence in correct English. Their responses, taped on a voice recorder, were transcribed later. Participants were not allowed to make notes, or listen to the sentences more than once. The reason for refusing to display the statements to participants was to direct their attention at meaning in order to elicit their implicit knowledge.

## 4 Results

In order to compare the impact of the recast types on the learners' implicit and explicit knowledge, a series of mixed between-within subjects analysis of variance (i.e., SPANOVA design) was run. As this statistical test does not provide post-hoc follow-ups, three one-way between-groups ANOVAs were separately conducted for each time period (pretests, immediate posttests and delayed posttests).

### 4.1 Implicit tests

The first research question investigated whether there were any significant effects of SR and MR on the acquisition of unreal conditionals, as measured by tests of implicit knowledge.

Table 2 details the descriptive statistics for the TGJT and the EOIT, representing learners' implicit knowledge for the three conditions across three time periods. Before proceeding with the main analysis, we checked whether our data conformed to the assumptions of this analysis. First, Levene's test of equality of error variance was conducted to make sure that the scores did not have unequal variances. The *p*-values of .123 and .215 indicated that this assumption was not violated. Then, the next assumption, Box's Test of Equality of Covariance Matrices was checked. The *p*-values (.004 & .002) turned larger than .001. Afterwards, the SPANOVA was run. The results are presented in Table 3 below:

**Table 2:** Descriptive statistics for measures of implicit knowledge (TGJT and EOIT).

tests	single recast			multiple recasts			control group		
	N	Mean	SD	N	Mean	SD	N	Mean	SD
TGJT Pretest	27	5.04	1.28	28	5.75	1.84	22	5.05	1.05
EOIT Pretest	27	1.67	2.09	28	1.82	1.22	22	.82	.91
TGJT immediate Posttest	26	9.50	1.50	27	10.11	.97	22	7.09	1.31
EOIT immediate Posttest	26	8.73	1.64	27	9.70	1.14	22	5.14	1.39
TGJT Delayed Posttest	26	9.19	1.57	26	9.73	1.04	22	6.95	1.36
EOIT Delayed Posttest	26	8.65	1.20	26	9.77	.992	22	5.82	1.26

**Table 3:** Multivariate tests (Wilks' Lambda).

Effect	Value	F	Error df	Sig.	Partial Eta Squared
TJGT Time * group	.65	8.21	138	.000	.19
EOIT Time * group	.62	9.40	138	.000	.21

* is an indication of the interaction effect.

Prior to viewing the main effects, we first need to assess the interaction effect, which indicates whether the changes in scores over time for the three conditions (SR, MR, and control group) have been statistically significant. This value for our data was *p* < .001, suggesting that the interaction effect was significant. The Multivariate Tests box indicates that the value for Wilks' Lambda for time was .65, *F*(4, 138) = 8.21, *p* < .05, partial eta squared = .19. As seen in Table 3, the treatments had a significant effect on the scores across time (test) periods. As for the EOIT, statistical differences were also found, *F*(4, 138) = 9.40, *p* < .05; Wilk's Lambda = .62; partial eta squared = .21. Therefore, we may conclude that group performance was significantly affected by the treatment that learners had experienced. There was also a significant interaction between the treatment and the posttest scores. Now that we have dealt with within-subjects effects, we may proceed to sift through the between-subjects effects.

As shown in Table 4, at the TGJT, the main effect comparing the two types of intervention has turned significant, *F*(2, 70) = 28.22, *p* < .001; partial eta squared = .47; in EOIT, the main effect comparing the two types of intervention was also significant, *F*(2, 70) = 73.78, *p* < .001; partial eta squared = .68. One-way ANOVAs were then run to compare the groups’ means on their implicit knowledge of grammar in order to probe the first null hypothesis.

**Table 4:** Tests of between-subjects effects.

Source	df	F	Sig.	Partial Eta Squared
TGJT	2, 70	28.22	.000	.47
EOIT	2, 70	73.78	.000	.68

The results in Table 5 suggest that, primarily, the model was significant for the TGJT; *F*(2, 72) = 36.93 *p* < .05. That is, the intervention had a significant effect on the subjects’ immediate and delayed posttest scores; *F*(2, 71) = 27.97 *p* < .05. Moreover, the effect size at the immediate posttest was found to be .50, and .44 at the delayed posttest respectively. In the EOIT, the results of the ANOVA for the immediate posttest indicated the existence of statistical difference(s); *F*(2, 72) = 69.44, *p* < .001, effect size = .65. Similarly, the results for the EOIT delayed posttest signified statistical difference(s); *F*(2, 71) = 43.69, *p* < .001, effect size = .55. Next, the multiple comparisons (Table 5) helped us find out where the differences lay*.*

**Table 5:** ANOVAs for implicit knowledge tests.

		Df	F	Sig.
TGJT Pretest	Between Groups	2	2.11	.12
	Within Groups	74		
EOIT Pretest	Between Groups	2	2.99	.06
	Within Groups	74		
TGJT Immediate Posttest	Between Groups	2	36.93	.000
	Within Groups	72		
EOIT Immediate Posttest	Between Groups	2	69.44	.000
	Within Groups	72		
TGJT Delayed Posttest	Between Groups	2	27.97	.000
	Within Groups	71		
EOIT Delayed Posttest	Between Groups	2	43.69	.000
	Within Groups	71		

Table 6 shows significant differences between the control and SR (*p* < .000), as well as between the control and MR conditions on the immediate TGJT (*p* < .000). However, between SR and MR (*p* = .196) no significant difference was observed. Furthermore, the delayed TGJT posttest showed a significant difference between the control group, and the SR and MR (*p* < .001), but once again, no such difference was found between the two experimental groups (*p* = .323). In sum, the post hoc test showed that the mean difference at the TGJT scores for the control group paled in comparison with those of the experimental groups, and that SR and MR have proved equally beneficial. In the case of the EOIT, the Tukey test illustrates that the mean score for the SR (M = 8.73, SD = 1.64) was significantly different from the control group (M = 5.14, SD = 1.39) on the immediate occasion. However, the MR (M = 9.70, SD = 1.14) fared significantly better than both the SR and the control groups. On the delayed EOIT, the MR (M = 9.77, SD = .992) was once again more effective than the control group (M = 5.82, SD = 1.25), and more importantly, than the SR (M = 8.65, SD = 1.20, p < .05).

**Table 6:** Multiple comparisons (Tukey test).

Dependent Variable	(I) group	(J) group	Mean Difference (I-J)	Sig.	95% Confidence Interval	
					Lower Bound	Upper Bound
immediate TGJT posttest	single recast	multiple recasts	-.611	.196	-1.45	.23
		control group	2.409*	.000	1.53	3.29
	multiple recasts	control group	3.020*	.000	2.14	3.90
immediate EOIT posttest	single recast	multiple recasts	-.973*	.04	-1.89	-.05
		control group	3.594*	.000	2.62	4.57
	multiple recasts	control group	4.567*	.000	3.60	5.53
delayed TGJT posttest	single recast	multiple recasts	-.538	.323	-1.43	.35
		control group	2.238*	.000	1.31	3.17
	multiple recasts	control group	2.776*	.000	1.84	3.71
delayed EOIT posttest	single recast	multiple recasts	-1.115*	.02	-2.11	-.13
		control group	2.836*	.000	1.80	3.87
	multiple recasts	control group	3.951*	.000	2.92	4.98

### 4.2 Explicit tests

The second research question examined whether there were any significant effects for SR and MR on the acquisition of unreal conditionals, as measured by tests of explicit knowledge.

Table 7 illustrates the descriptive statistics for the UGJT which represented explicit knowledge of the learners across three time periods. Before proceeding to the analysis of the results, the assumptions underlying multivariate ANOVA were checked. A mixed between-within subjects analysis of variance was then conducted to assess the impact of treatments on the participants' performance at the UGJT. The Multivariate Tests indicated that Wilk's Lambda = .57, *F*(4, 138) = 10.82, *p* < .05, partial eta squared = .20. That means that an increase in test scores across the time periods has been identified and the interaction effect is statistically significant. Moreover, tests of between-subjects effects signified that the main effect has also turned significant, *F*(2, 70) = 31.01, *p* < .001; partial eta squared = .47.

**Table 7:** Descriptive statistics for measure of explicit knowledge.

UGJT	Group	N	Mean	SD
pretest	single recast	27	5.52	1.45
	multiple recasts	28	6.08	1.84
	control group	22	6.00	1.19
immediate posttest	single recast	26	10.00	1.55
	multiple recasts	27	10.48	1.01
	control group	22	7.50	1.14
delayed posttest	single recast	26	9.73	1.66
	multiple recasts	26	10.46	1.33
	control group	22	7.14	1.32

The results of a series of one-way ANOVAs run to compare group differences at the UGJT (Table 8) were indicative of statistical differences in the immediate posttest: *F*(2, 72) = 37.89, *p* < .001, with an effect size of .51, as well as in the delayed posttest, *F*(2, 71) = 33.66 *p* < .05, with an effect size of .48.

**Table 8:** One-way ANOVAs for UGJT.

Test		df	F	Sig.
pretest	Between Groups	2	.99	.38
	Within Groups	74		
immediate posttest	Between Groups	2	37.89	.000
	Within Groups	72		
delayed posttest	Between Groups	2	33.66	.000
	Within Groups	71		

Post-hoc comparisons (Table 9) indicate that the mean score for the SR (M = 10, SD = 1.55) was significantly different from the control group (M = 7.50, SD = 1.14). The MR (M = 10.48, SD = 1.01) was similarly different from the control group (M = 7.50, SD = 1.14). However, no significant difference was found between the two experimental groups. That is, the experimental conditions uniformly performed better at the explicit knowledge test.

**Table 9:** Multiple comparisons (Tukey test).

Dependent Variable	(I) group	(J) group	Mean Difference (I-J)	Sig.	95% Confidence Interval	
					Lower Bound	Upper Bound
immediate UGJT posttest	single recast	multiple recasts	-.481	.35	-1.31	.35
		control group	2.500^∗^	.000	1.63	3.37
	multiple recasts	control group	2.981^∗^	.000	2.12	3.85
delayed UGJT posttest	single recast	multiple recasts	-.731	.17	-1.70	.23
		control group	2.594^∗^	.000	1.59	3.60
	multiple recasts	control group	3.325^∗^	.000	2.32	4.33

## 5 Discussion

The main purpose of this research was to examine the differential effects of two recasting strategies on learning a newly presented target structure as reflected on the implicit and explicit knowledge of EFL learners. As Sheen (2006) also confirmed, it is often the case that, novice researchers proceed with a general definition of recast, making little attempt to investigate its subcategories in accordance with its diverse functions. This study attempted to address the pedagogical dilemma of what recast type to prioritize in the L2 classroom, an issue that teachers may have little awareness of. The results from the EOIT, TGJT, and UGJT revealed that recasts increased learners’ retention of unreal conditionals on both immediate and delayed posttests. These findings, in principle, support the theoretical claims made in favor of recasts (e.g., Goo and Mackey, 2013; Li, 2013; Long, 2007; Mackey, 1999; Mackey and Goo, 2007; Mackey and Philp, 1998).

To address the first research question as to how recasts would affect learners’ implicit knowledge, the EOIT scores revealed that the MR condition ranked the highest at both immediate and delayed tasks, with the SR trailing behind. This was not the case at the TGJT where both experimental groups equally and analogously improved from the pretest to the immediate and delayed posttests. This suggests that both recast types have proved effective, with MR even edging ahead at the EOIT. In retrospect, a number of studies (e.g. Lyster and Saito, 2010; Nassaji, 2017; Révész, 2012) found that recasts were more strongly represented on oral than written tasks. This may be attributable to transfer appropriate processing (Lightbown, 2008), which suggests that L2 learning is contingent upon context; what is learned in one context will be more easily retrieved during similar cognitive processes. As recasts in this study were orally presented, the experimental groups had a correspondingly easier time on the EOIT.

**Fig. 1:**
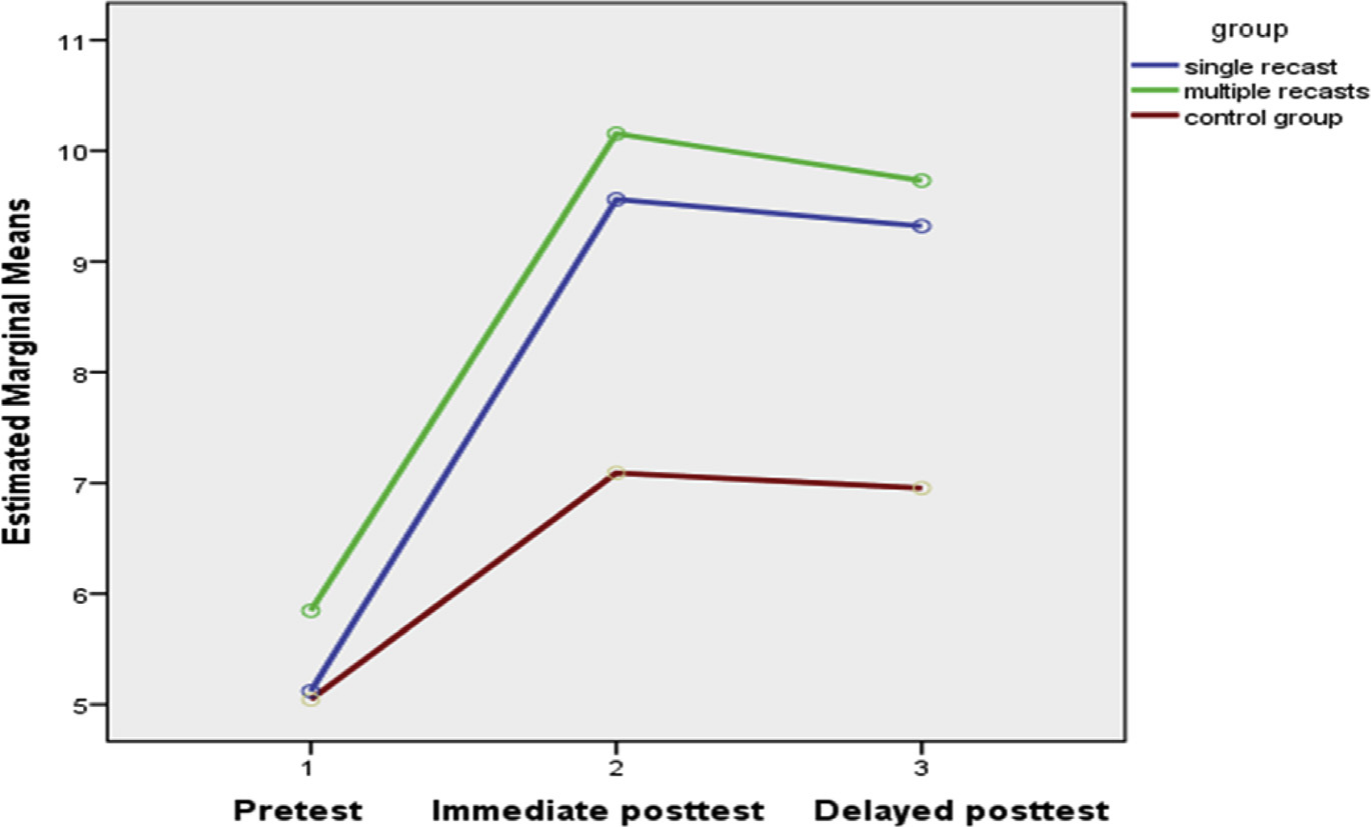
TGJT accuracy scores.

**Fig. 2:**
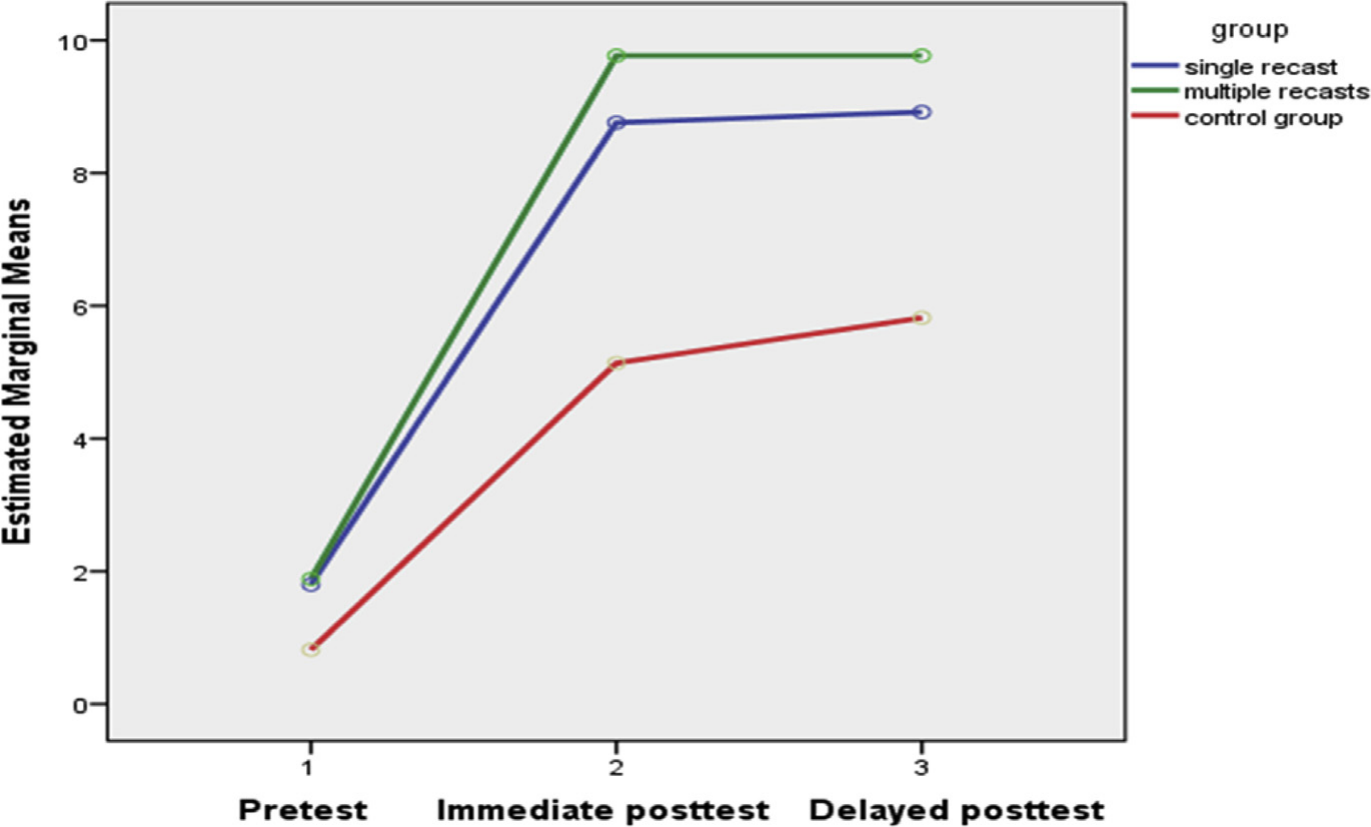
EOIT accuracy scores.

**Fig. 3:**
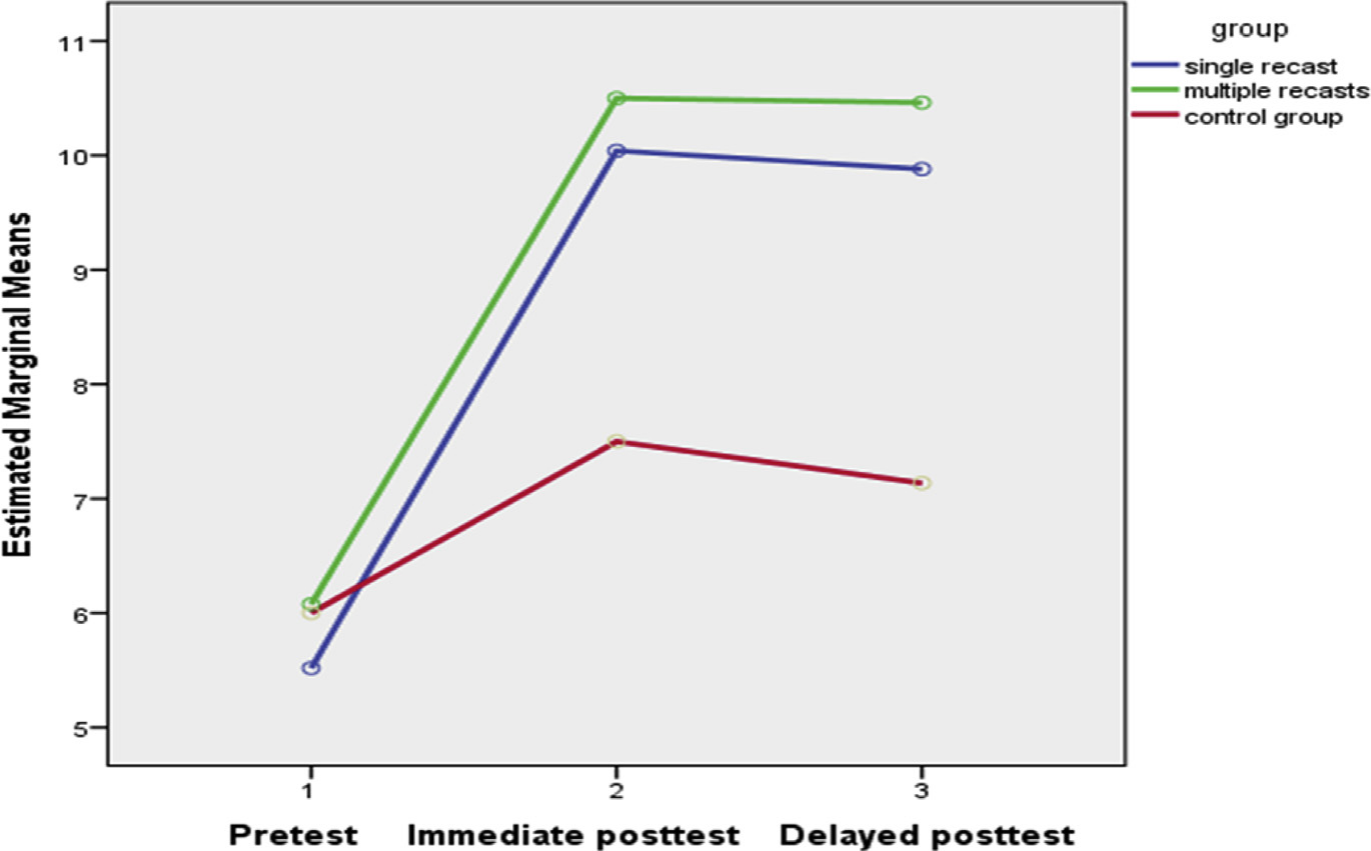
UGJT accuracy scores.

On the second research question regarding the impact of recast types on explicit knowledge, the UGJT findings revealed that the control group failed to represent any improvement on the immediate and delayed posttests. Thus, recasts were facilitative in the acquisition of explicit knowledge as well. The results suggest that the effect of recasting on the learners’ explicit knowledge may have been more consistent than on their implicit knowledge. In contrast to what we found, Li (2010) had observed that recasts may be more beneficial for implicit knowledge only. As illustrated by Figs. 2 and 3, at the EOIT and UGJT both experimental conditions fared well from the immediate to the delayed posttests, but this was not the case for the TGJT (see Fig. 1) where experimental groups' retention fairly declined at the delayed tests.

Single recasts are implicit in essence. It is argued that implicit recasts are an ideal type of CF, because the correct form they provide can stand in contrast to the learner's erroneous output, resulting in a cognitive comparison (Doughty, 2001). In fact, single recasts prevent from disrupting the communication flow, such that learners' cognitive resources can be distributed in an effective way, and attention can be simultaneously directed to meaning and form (see Long, 2007). Furthermore, the learners receiving MR had a strong performance on implicit and explicit knowledge tests. Apparently, multiple recasts stand a higher chance of being noticed. As denoted by Nassaji (2009), they are more salient and more likely to be attended to. Hence, they appear more likely to facilitate L2 learning on grounds of saliency which is explicable in terms of Schmidt's noticing hypothesis (1990). However, only few studies have investigated whether explicitness in recasts is crucial to acquisition. These two different yet related types of knowledge, require additional examination in SLA research since different types of instruction may arouse different patterns of knowledge development. It must be acknowledged that although we attempted to draw a distinction between these two knowledge types, in reality, as Ellis et al. (2009) observed, it is difficult to set them apart, as learners may employ both knowledge types at their disposal interchangeably and in various degrees during task completion.

Taken together, the impact of recasts on both implicit and explicit knowledge did not differ much regardless of the feedback type (single or multiple) provided. In other words, contrary to a number of studies (e. g., Egi, 2010; Li, 2010; Lyster, 1998a, Lyster and Ranta, 1997; Panova and Lyster, 2002) that suggested learners may not notice the corrective purpose of recasts, the current data demonstrated that their effectiveness on explicit and implicit knowledge is unequivocal. The findings also square with Erlam and Loewen (2010) who found no disparity between explicit and implicit conditions. As for the delayed effect, on all tasks, the differential effects of the recasts persisted over time. Several previous studies had reached similar outcomes on recasts’ delayed retention (Ellis et al., 2006; Loewen and Nabei, 2007; Nassaji, 2017; Révész, 2012). One contribution of recasting to L2 learning is the provision of both positive and negative evidence. Unlike Doughty (2001), Long (2015), and Goo and Mackey (2013), who argued that the corrective function of recasts is too implicit to be understood by learners (positive evidence), the current results indicated that SR could go a long way towards achieving this aim. A number of studies (e.g. Doughty, 2001; Long, 2007; Saxton, 2005) had observed that recasts raise attention on the part of the learners, and owing to their reactive nature, they do not stop the flow of communication. Hence, they are considered to be more communicatively helpful than other types of explicit CF that postpone the current of conversation. In reality, recasts can be made more effective in promoting L2 knowledge when learners are able to notice the corrective focus through additional oral techniques similar to those applied in the present study.

### 5.1 Implications

The current research has only attempted to add one more building block to the recent contributions towards strengthening the interface between CF and L2 grammar research. Despite its exploratory nature, the data obtained can be interpreted as shedding further light on the learning potential that may derive from applying different forms of CF on different grammatical forms. The findings carry strong reasons for SLA researchers and L2 teachers for claiming that recasting is beneficial in developing EFL students' grammatical accuracy in classroom settings. On a broader scope, this study might bear pedagogical implications for L2 teaching such that language production tasks can be more high-yielding if they are practiced collaboratively on the part of the novice (L2 learner) and the expert (teacher). This collaborative production leads learners to reason about the correct use of language forms, thereby increasing their awareness of meaning connections (see Izumi, 2002). Pedagogically speaking, L2 teachers ought to gain a more conscious recognition of the effectiveness of more and less implicit recasts, despite the consensus among researchers that recasts would not necessarily lead to acquisition.

### 5.2 Limitations

As any other classroom-based research, this study suffered limitations including in the design of the study, though the payoff was of high ecological validity. One major setback lay with the shortage of resources across typical language institutes for data collection purposes. Rarely are researchers at liberty to take over the reins for classroom handling decisions. Administering the EOIT would ideally require a well-equipped laboratory where all participants could simultaneously listen to the audio and record their voices. The researcher however, had to take the trouble to perform this painstaking and time-consuming task in a classroom context. Moreover, for some students, the EOIT was much of a demanding task. They requested the researcher to play the audio tracks more than once, as they were occasionally unable to comprehend certain language chunks. In addition, the results may have been contaminated by test effect since scores of the EOIT for the control group increased on the immediate and delayed posttests. As all the statements in the EOIT were the same in content but different in order, the participants in the control group could become familiar with the task content on the immediate and delayed posttests. This drawback could have been minimized if there had been three different counterbalanced tests. Moreover, inevitably, the control group went on with their regular classes at the institute over and above what was scheduled for them during the treatment period.

## 6 Conclusions

Overall, the findings highlighted the prominent role of SR and MR as prevailing modes of effective CF in classroom settings on the attainment of conditionals. A potentially noteworthy issue which deserves further attention, is the verification and re-evaluation of L2 implicit and explicit knowledge measures. While the current study, established its measurement procedure on the basis of previous research; that is, using several well-known instruments to tap into implicit and explicit knowledge, the validation of such measures warrants further investigation particularly with regard to the participants and instructional settings. The outcome discrepancy between the two implicit knowledge measures testifies to a certain degree of measurement imprecision existing therein. For further reading, we suggest a thorough review of implicit knowledge measures by Kim and Nam (2017).

A viable theme that could be considered in future research is the role of individual learner differences in predicting the success of single and multiple, or other recasting strategies, which was not attended to at this study. Li (2013), for instance, discovered a relationship among the effect of recasts, grammaticality judgment tasks, and learners' language analytic ability. One question that may arise here is whether learners’ language analytic ability would also play a role in predicting the differential effects of single and multiple recasts. Thus, it would be worthwhile to explore whether more analytic learners are more capable than less analytic learners of tuning their attention to a specific language form in a context where errors arise incidentally. Another issue of concern is that of the uptake (learner responses to corrective feedback), which should be the object of inquiry and assessment in future CF research. A topic of potential interest for L2 researchers is to draw more solid conclusions about the long-term effects of recasts. In so doing, longitudinal studies examining the delayed effects over a longer period of time and in a different context are needed. The findings about the delayed effects of recasts should be interpreted in terms of how such outcomes are defined and measured. In this study, as conventionally practiced, delayed effects were measured by tests that were identical to the immediate posttests and were also administered after a short interval following the treatment (one week). Although the results of such tests may show that recasts help learners to maintain the target forms during that interval, they can hardly indicate whether learners would incorporate the forms into their long-term interlanguage system. Furthermore, they do not show whether learners have the ability to transfer their knowledge to more naturalistic or communicative contexts.

## Declarations

### Author contribution statement

Mohammad Hassanzadeh: Conceived and designed the experiments; Analyzed and interpreted the data; Contributed reagents, materials, analysis tools or data; Wrote the paper.

Fahimeh Marefat: Analyzed and interpreted the data; Wrote the paper.

Arezoo Ramezani: Performed the experiments; Wrote the paper.

### Funding statement

This research did not receive any specific grant from funding agencies in the public, commercial, or not-for-profit sectors.

### Competing interest statement

The authors declare no conflict of interest.

### Additional information

No additional information is available for this paper.
